# Editorial for the Special Issue on Advances in Capacitive Sensors

**DOI:** 10.3390/mi11110993

**Published:** 2020-11-05

**Authors:** Juan A. López-Villanueva, Almudena Rivadeneyra

**Affiliations:** Departamento de Electrónica y Tecnología de Computadores, Facultad de Ciencias, Campus Universitario de Fuentenueva, University of Granada, 18071 Granada, Spain

Capacitive sensors are an active research area with multiple advantages and great applicability. Their numerous benefits, such as simple structure, cost-effectiveness, low power consumption, low noise performance, reliability, and high adaptability to the environment, have made them very suitable for the measurement of many physical and chemical magnitudes. They are used in a wide variety of applications, from tactile sensors, flexible human–computer interfaces, artificial muscles, wearable systems and electronic skin to material property monitoring, food inspection, or biological sensing, among others. Detection mechanisms can be based on changes in the sensor geometry or in variations of the dielectric properties of a sensing layer. Therefore, in addition to many proposals of new innovative geometries, there is also much research work on advanced materials and nanostructures based on them, as well as on fabrication technologies, such as those based on microsystems or additive techniques. All of these aspects make capacitive sensors a very interdisciplinary research area that motivates launching this Special Issue on Advances in Capacitive Sensors, with the aim of collecting papers from structures or systems based on novel geometries, fabrication procedures, innovative materials, and even measurement techniques. This Special Issue includes five original research papers, representative of the various aspects mentioned above, and a review paper.

The fabrication aspects are covered in two papers devoted to two of the most advanced techniques for fabrication of sensors and microsystems, MEMS and printed, in which novel devices are also demonstrated. In the MEMS case, K. Rao et al. present a micromachined micro-g capacitive accelerometer based on a through-silicon wafer-etching process, with a silicon-based spring-mass sensing element, in which the displacement changes of the proof mass are sensed by an area-variation-based capacitive displacement transducer [1]. The accelerometer prototype is fabricated and characterized, and it appears as a promising solution to be used for inertial navigation, structural health monitoring, and tilt measurement applications. As a printed electronics application, F.J. Romero et al. present a simple, fast, and cost-effective method for the large-scale fabrication of high-sensitivity humidity sensors based on graphene-oxide (GO) and poly(3,4-ethylenedioxythiophene) polystyrene sulfonate (PEDOT:PSS) composites on flexible substrates [2]. The results show that the presence of the PEDOT:PSS within the GO structure is able to modify the electrical properties of the sensitive film, improving the overall performance of the sensors. This technology may be considered a step forward in the cost-effective fabrication of high-performance small flexible sensors.

Concerning special device structures and measurement issues, there are another two papers related to capacitive sensors for spherical joints employed as multiple degree-of-freedom mechanical hinges in some engineering applications, e.g., robots and parallel manipulators. W. Wang et al. present a novel approach for rotational angle measurement of precision spherical joints with a capacitive sensor [3]. The mathematical model for the rotational angles of a spherical joint and the capacitance are deduced, and the capacitance values of the capacitors at different rotations are simulated, suggesting the feasibility and effectiveness of the proposed capacitive detection method. The problem of detecting the micro-clearance in spherical joints, for which capacitive sensors are a very interesting solution, is also considered [4]. Five error sources of spherical capacitive sensors are examined, and their mathematical models are deduced and validated by simulation. Compared with other methods, capacitive sensors have the advantages of high resolution and good dynamic performance for this type of systems.

In the measurement circuit and systems field, Y. Xie et al. model and analyze the noise performance of a capacitive sensing circuit with a differential transformer with potential application in space-borne gravitational wave detection [5]. This is an application that demands very high sensitivity, especially in the wave detector considered in this paper, which has a much shorter arm length compared to other twell-known gravitational-wave detectors. The transfer function and capacitance measurement noise of the circuit are modeled and analyzed, and figures of merit are proposed to optimize the measurement resolution of the circuit. As a fundamental component of the measurement circuit, the transformer has been fabricated and evaluated, showing that it can generate a sufficient capacitance resolution to satisfy the stringent requirements of this application.

Finally, A. Rivadeneyra et al. conclude the Issue with a general review on recent advanced in printed capacitive sensors, focused on printing techniques [6]. The latest advances in the field of capacitive sensors fabricated by printing techniques are summarized and the main fabrication technologies used in printed electronics are explained, pointing out their features and uses, and discussing their advantages and drawbacks. The main types of capacitive sensors manufactured with different materials and techniques from physical to chemical detection are detailed, as well as the measured ranges. The paper concludes with a short notice on status and perspectives in the field.

We hope that this Special Issue on Advances in Capacitive Sensors will offer readers a good overview of the current state of the art with interesting illustrative examples in different aspects of this field.
